# Bacterial-fungal metabolic interactions within the microbiota and their potential relevance in human health and disease: a short review

**DOI:** 10.1080/19490976.2022.2105610

**Published:** 2022-07-28

**Authors:** Alexia Lapiere, Mathias L Richard

**Affiliations:** a Université Paris-Saclay, INRAE, AgroParisTech, Micalis Institute, Jouy-en-Josas, France; b Paris Center for Microbiome Medicine, Fédération Hospitalo-Universitaire, France

**Keywords:** Microbiota, bacteria, fungi, interaction, metabolites, interkingdom, human health, disease

## Abstract

The composition of the microbiota is the focus of many recent publications describing the effects of the microbiota on host health. In recent years, research has progressed further, investigating not only the diversity of genes and functions but also metabolites produced by microorganisms composing the microbiota of various niches and how these metabolites affect and shape the microbial community. While an abundance of data has been published on bacterial interactions, much less data are available on the interactions of bacteria with another component of the microbiota: the fungal community. Although present in smaller numbers, fungi are essential to the balance of this complex microbial ecosystem. Both bacterial and fungal communities produce metabolites that influence their own population but also that of the other. However, to date, interkingdom interactions occurring through metabolites produced by bacteria and fungi have rarely been described. In this review, we describe the major metabolites produced by both kingdoms and discuss how they influence each other, by what mechanisms and with what consequences for the host.

## Introduction

The human body hosts multiple microbiota, composed of several types of microorganisms, mainly bacteria but also fungi, archaea and viruses. All these populations live together and form complex and dynamic ecosystems. Due to their multiple functions, microbiota are considered indisputable actors in human health. Bacteria, as major microorganisms observed in human microbiota, are widely studied and have been associated with homeostasis maintenance and pathology development.^1^ Fungal communities, although present in lower abundance than bacteria, also play an important role in health and disease.^2^ Nevertheless, the fungal population of the microbiota is still little studied, for different reasons but mainly because there is a smaller scientific community involved in these topics and because the databases of whole genomes of fungi are less provided. In addition, there are technical problems such as under-representation of DNA in samples and contamination with eukaryotic DNA of the host. As part of an ecosystem, microorganisms found in the microbiota naturally interact with each other. In recent years, particular attention has been given to interspecies bacterial interactions; however, bacteria and fungi are present in the same niches and are also able to with each other. Moreover, several studies have linked specific bacterial-fungal interactions to disease development/severity.^3^ These bacterial-fungal interactions can be antagonistic, typically illustrated by competition for niche colonization and nutrient sources, or synergistic, for example, by cooperating for mutual growth.^4^ Among the interactions observed in mixed biofilms, the literature demonstrates the significance of metabolite-driven communication on human health. Metabolic interactions between bacteria and fungi involve a wide range of primary and secondary metabolites. Primary metabolites, such as alcohols, organic acids, amino acids and vitamins, are directly involved in biological processes that are vital to cells. Siderophores, quorum sensing molecules and fatty acids are secondary metabolites that are not essential but contribute to important cellular functions. Correspondingly, through these multiple metabolites, bacteria and fungi induce various effects on each other: enhancement or inhibition of growth and/or morphogenic transition and changes in gene expression or metabolism, which affect many aspects of the microorganism’s development that may contribute positively or negatively to human health; we will discuss and describe these interactions in this review.

## Metal siderophores – key factors of intermicrobial interactions

Metals are essential elements for eukaryotic and prokaryotic organism survival. Among existing metals, iron is one of the most important for microorganisms due to its involvement in several vital biological processes (respiration, biomolecule synthesis, etc.).^5^ A high iron concentration is toxic to cells, as iron is associated with free radical formation, which causes injuries to cellular components.^6^ Therefore, to maintain low levels in the organism, the mammalian host regulates the free iron concentration. This control of iron availability by the host is also associated with the phenomenon termed “nutritional immunity”; by modulating nutrient availability for microorganisms, the host prevents pathogen overgrowth.^7^ In such an iron-limited environment, bacteria and fungi had to develop strategies to uptake this metal. Microorganisms generally use various iron uptake pathways,^8,9^ with two major relevant pathways. The first pathway, called the reductive pathway, consists of iron reduction from ferric to ferrous form (Fe^3+^ to Fe^2+^) by the action of metallo-reductases found on the cell membrane. The ferrous form is then assimilated by the cell. A second pathway used by microorganisms is the nonreductive pathway. This process requires specific secreted molecules called siderophores, which are necessary to scavenge iron in the environment and are then absorbed by the body. Due to the high affinity between iron and siderophores and between siderophores and membrane-specific receptors, the nonreductive pathway facilitates iron absorption by microorganisms in an iron-limited environment. Different siderophore types have been described, such as hydroxamate, catecholate, phenolate, and carboxylate, based on their mechanisms of iron complexification, as each siderophore type requires a specific iron transporter (SIT) to be assimilated through the organism membrane.^10^

While nearly all bacteria and fungi possess SIT to uptake siderophores, they do not all possess the siderophore synthesis ability. For example, the yeast *Saccharomyces cerevisiae* is able to assimilate siderophores, but its genome does not show any genes coding for siderophore synthesis.^11^ Consequently, *S. cerevisiae* uses exogenous siderophores, named xenosiderophores, secreted by neighboring fungal or bacterial organisms.^12^ Genomic analysis of other fungi has also revealed a lack of siderophore synthesis genes, particularly in *Candida albican*s and *Cryptococcus neoformans*, which thus participate as well in the phenomenon called “iron parasitism” by using xenosiderophores.^13^ Each microorganism exhibits specific siderophore receptors, reflecting a specificity for siderophore types and a distinct technique of iron uptake. *C. albicans* harbors only one Arn1/Sit1 transporter, which is involved in catecholate siderophore uptake,^14^ while *S. cerevisiae* has four siderophore membrane transporters, namely, Arn1p, Sit1p/Arn3p, Taf1p/Arn2p and Enb1p/Arn4p, which each have a specificity toward a siderophore type (hydroxamate, catecholate, etc.) or a specific siderophore (i.e., Enb1p/Arn4p is specific for bacterial enterobactin).^8^ Siderophore transporters are, however, not specific for a single species since it has been observed that various strains of *S. cerevisiae* exhibit individual transporters.^11^ Bacteria can also take advantage of their fungal counterparts for iron assimilation. *In vitro* studies have shown that mutant *Escherichia coli* deficient in siderophore synthesis (enterobactin) are able to use fungal hydroxamate-type siderophores, such as ferrichrome and coprogen secreted by *Penicillium spp*., through the Fhu system.^15^ Moreover, in the presence of *Penicillium spp., E. coli* enterobactin production and uptake genes are upregulated, which suggests that *E. coli* can use its native siderophores and xenosiderophores at the same time, illustrating flexibility toward siderophore availability.

Social interactions between microorganisms shape their evolutionary changes and their dynamics. From the perspective of “iron parasites”, such as *S. cerevisiae*, C.C. Philpott^12^ suggested that yeast evolution in a wide microbial community and a siderophore-rich environment could have resulted in the use of xenosiderophores without secretion. Two hypotheses could possibly explain this phenomenon: (i) the siderophore-rich environment has resulted in genomic changes, such as the loss of siderophore synthesis genes, considering that neighboring microorganisms could provide them; or (ii) new/novel functions related to siderophore uptake and transport mechanisms have been acquired. Either hypothesis shows that *S. cerevisiae* takes advantage of the microbial community for iron assimilation and that this metal plays a major role in microbial interactions. As siderophore synthesis is an energy-consuming phenomenon for the producer microorganism, “parasitized” microorganisms react by cooperation or competition for iron uptake in the presence of siderophore nonproducers. For instance, *Pseudomonas aeruginosa* responds differentially to siderophore nonproducer bacterial counterparts, depending on the nonproducer microorganism and on the iron availability in the environment.^16–18^

Several bacterial-fungal interactions involving iron metabolism are observed in the clinic, mostly at disease onset. Generally found in respiratory airways of cystic fibrosis patients, the fungus *Aspergillus fumigatus* and the bacterium *P. aeruginosa* are associated with serious pulmonary infections.^19,20^ In pulmonary microbiota, these two organisms use iron metabolism to compete with each other. Therefore, iron plays a significant role in mixed pulmonary infection with both organisms. *P. aeruginosa* secretes pyoverdine, a specific siderophore that *A. fumigatus* cannot assimilate.^21^ Thus, pyoverdine sequesters iron from *A. fumigatus*, affecting its growth. However, *A. fumigatus* responds to this iron competition by producing its own hydroxamate siderophores, which *P. aeruginosa* is unable to use.^21^ Mutant *A. fumigatus* defective in hydroxamate siderophore production were more susceptible to pyoverdine. *P. aeruginosa* also has an antagonistic relationship linked to iron with *C. albicans*, another opportunistic fungus. The bacterium not only sequesters iron from *C. albicans* but also kills the yeast, which might be a way to use its intracellular iron.^22^ However, to counteract *P. aeruginosa, C. albicans* is able to repress pyoverdine expression and to then attenuate *P. aeruginosa* virulence as a result.^23^ Microbial interactions involving iron indirectly impact microorganism virulence. *C. albicans* and *A. fumigatus* pathogenicity is considerably affected by iron availability in the environment and the organism’s ability to uptake iron. In an iron-limited environment, *C. albicans* activates genes involved in iron uptake, which also trigger virulence genes.^24,25^ *A. fumigatus* virulence is associated with its siderophore secretion ability, as a mutant for siderophore synthesis exhibits reduced virulence.^26^

As it is the subject of most of the articles focused on metal metabolism-based interactions between bacteria and fungi, iron is the main metal studied in this section. However, the family of metals includes other biologically essential components, such as zinc, copper, manganese and nickel. Little is currently known of bacterial-fungal interactions involving these metals. To date, no article has demonstrated that these metals might play an equivalent role in interkingdom interactions. However, their role in microorganism biological processes is undeniable, notably zinc, manganese and copper, which are necessary for SOD enzyme production, a family of enzymes essential for microorganism detoxification of reactive oxygen species.^27,28^ It has been shown that nonreductive uptake pathways exist for some of these metals. Microorganisms are able to secrete molecules called zincophores, chalkophores and nickelophores for zinc, copper and nickel scavenging in the environment, respectively.^29,30^ We can then hypothesize the microbial interactions around these molecules, as observed with iron and siderophores. Findings have also demonstrated that iron siderophores are able to link with Cu and Zn ions,^30^ suggesting that shared siderophores could involve not only iron but also several other metals and thus impact the interactions between fungi and bacteria. Continuing the exploration of the role of metals in microbial interactions seems to be essential, considering that these interactions around metals are closely linked to human health and the virulence of specific microorganisms.

## Quorum sensing – a prominent means of interkingdom communication

To adapt to their environment, bacterial and fungal communities had to develop specific molecular crosstalk to communicate with their species counterparts. The phenomenon termed quorum sensing (QS) designates a distinct form of communication between cells of a single species.^31^ It corresponds to a metabolic pathway in which microorganisms send chemical signals to establish a collective behavior, depending on various factors such as nutrient availability and adhesion site vacancy, therefore creating a coordinated response against other competitor organisms or simply regulating their own population for better survival.^32^ Microbes sense their own population density with quorum-sensing molecule (QSM) accumulation. When a specific level of QSMs is reached, a collective change in gene expression occurs, inducing a modification of the growth mode; morphological transition, biofilm formation and virulence factor expression are generally affected.^33^

When QS was discovered, this phenomenon was first associated with the bacterial population only. Various types of QS bacterial signals have been identified, such as acylhomoserine lactone, diffusible signal factors, and autoinducer 2,^34^ that are each specific to a bacterial type. Recently, it was observed that *C. albicans* is able to secrete a QSM named farnesol, the first identified eukaryotic organism QSM, which is able to inhibit *C. albicans* yeast to hyphae morphogenesis.^35^ After this finding, it became clear that QS also plays a major role in fungal communities. Further studies showed that other fungi secrete QSMs as well, such as *Aspergillus nidulans* and *C. neoformans*.^36,37^ Moreover, since bacteria and fungi generally coexist in several ecosystems, QS signal interaction is evidently an additional method of communication between these organisms. Interkingdom QS interaction has only been demonstrated recently with the discovery of the *P. aeruginosa* QSM effect on *C. albicans* morphogenesis.^38^ The *P. aeruginosa* QSM 3-oxo-C12 homoserine lactone (3-oxo-C12-HSL) shows molecular similarities with the *C. albicans* QSM farnesol. Based on this observation, it has been determined that 3-oxo-C12-HSL acts on the same Ras1-cAMP-Efg1 pathway as farnesol to inhibit *C. albicans* morphogenesis.^39^ The QS interaction between *C. albicans* and *P. aeruginosa* works bidirectionally, as farnesol affects *P. aeruginosa* by inhibiting cell motility and the production of pyocanin, a toxic molecule for *C. albicans*.^40,41^ Since the discovery of the role of QS in interkingdom dialog, various bacterial-fungal QS interactions have been described.

The findings on the *P. aeruginosa* 3-oxo-C12-HSL effect on *C. albicans* growth have led the way for several studies on other bacterial QSM effects on fungi. Various fungi have shown impaired growth following *P. aeruginosa* QSM exposure. *A. fumigatus* exhibits altered biofilm formation and conidial germination in the presence of *P. aeruginosa* 3-oxo-C12-HSL,^42^ and *Cryptococcus* spp. display growth inhibition in a medium containing PQS (2-heptyl-3,4-dihydroxyquinoline), another extracellular QS signal produced by *P. aeruginosa*.^43^ Several other bacteria specifically affect *C. albicans* filamentation. *Burkholderia cepacia* BDSF (Burkholderia Diffusible Signal Factor), also called cis-2-dodecenoic acid, is able to suppress the *C. albicans* yeast-to-hyphae transition.^44^ The underlying mechanism of BDSF is still unknown; however, it has been shown that it is functionally different from the farnesol and 3-oxo-C12-HSL operating mode and it is suggested that its effect operates through the Sfl1 pathway.^45^ Competence stimulating peptide (CSP), a QSM secreted by *Streptococcus mutans*, inhibits *C. albicans* hyphal morphogenesis.^46^ However, if other *Streptococcus* species have shown a similar inhibitory effect on *C. albicans*, one strain of *S. gordonii* stimulates *C. albicans* filamentation by the secretion of a widespread bacterial QSM, autoinducer 2.^47^ Interestingly, another autoinducer 2 producer, *Aggregatibacter actinomycetemcomitans*, demonstrated opposite results on *C. albicans* morphogenesis.^48^ These results are intriguing, since autoinducer 2 is considered a universal bacterial signal, with a preserved structure among bacteria.^34^ In their review, Dixon and Hall suggested paths of reflection to understand this difference in the autoinducer 2 effects on *C. albicans*.^34^ Either the studies have variations in their protocols, i.e., *Candida* strains and culture medium, or *C. albicans* have the ability to distinguish between autoinducer 2 molecules produced by different bacterial species, considering that *C. albicans* generally cohabits with various bacteria and might have evolved a strategy to discriminate them. However, such a strategy in yeast has not yet been investigated.

The impact of fungal QSMs on bacteria has also been reported in several studies. In addition to the *P. aeruginosa* inhibitory effect described above, farnesol has also shown antagonistic interactions with other bacteria. Jabra-Rizk *et al*. demonstrated in two distinct articles the concentration-dependent effect of farnesol on *Staphylococcus aureus*. In their first publication, they observed that a high concentration of farnesol reduces *S. aureus* survival by impairing biofilm formation and damaging the cell membrane.^49^ The use of a high concentration of farnesol (>100 µM) also induced an increase in *S. aureus* sensitivity to antimicrobial compounds. However, in their second publication, a moderate level of farnesol (40–50 µM), as observed in *C. albicans* biofilms, enhanced *S. aureus* tolerance to the same antimicrobial compounds previously tested.^50^ It is interesting to note that farnesol, depending on its concentration, induces the opposite results. This observation is important, as farnesol is considered for therapeutic use against pathogenic bacteria. Therefore, close attention is needed to the concentrations used during clinical trials to obtain the expected beneficial impact of farnesol. In another study, Peleg *et al*. demonstrated a farnesol-driven defensive response of *C. albicans* to *Acinetobacter baumannii* in a nematode *Caenorhabditis elegans* coinfection model.^51^ Following nematode coinfection, *A. baumannii* induces inhibition of *C. albicans* filamentation. However, *in vitro* experiments highlighted the antibacterial activity of the *C. albicans* supernatant on *A. baumannii*, and this antibacterial effect was higher as the *C. albicans* biofilm matured. The team hypothesized that farnesol was involved in *A. baumannii* growth inhibition and demonstrated that the supernatant of mutant *C. albicans*, defective in farnesol production, caused no inhibition, supporting the role of farnesol in the *C. albicans* counteroffensive toward *A. baumannii*. Finally, tyrosol, another fungal QSM generally associated with *Candida* spp., also impacts bacterial activity. In addition to promoting *C. albicans* filamentation, tyrosol has been found to inhibit the *P. aeruginosa* production of the virulence factors hemolysin and protease^52^ and *S. mutans* biofilm formation, hypothetically through disruption of cell integrity.^53^

QS appears to be a complex, intricate system of communication involving a wide range of actors; however, the existence of QS inhibitors further complicates this system. Indeed, if QSMs are involved in bacterial-fungal metabolic communication, quorum quencher molecules from bacteria and fungi are able to interfere with the QS dialog. Various mechanisms of quorum quenching have been reported: inhibition of QS molecule synthesis, inhibition of QS molecule-receptor interaction and modification/degradation of QSMs.^54^ Recently, a study demonstrated a QS inhibitory molecule produced by kefir fungi against pathogenic gut bacteria:^55^ *Kluyveromyces marxianus* is a predominant yeast found in kefir, a fermented beverage considered a probiotic food with therapeutic benefits for health. *K. marxianus* is able to secrete high quantities of tryptophol, which has shown an inhibitory effect on the biofilm production of *Vibrio cholerae*, a deleterious bacterium in the human gut. Through tryptophol secretion, *K. marxianus* interferes with *V. cholerae* autoinducer CAI-1-driven QS communication, resulting in decreased biofilm production and thus reduced virulence. To date, this study is the only one demonstrating a bacterial-fungal interaction involving quorum quenching in the human ecosystem. Other bacterial-fungal quorum quenching has been observed in environmental ecosystems (soil, mycorrhiza, and water), i.e., root-associated fungi, belonging to the *Ascomycota* and *Basidiomycota* lineages, are able to secrete lactonase, an enzyme that deteriorates the bacterial N-acyl homoserine lactone QSM.^56^ It is thought that fungi and bacteria found in human microbiological niches could be able to secrete quorum quenching lactonases, considering that they have evolved together in the same ecosystem and developed sophisticated interkingdom interactions, including QS. However, no study has explored this hypothesis at this time.

## Effects of vaginal bacterial organic acids on *Candida* species

The vaginal microbiome hosts both bacterial and fungal organisms. Most predominant vaginal bacteria belong to *Lactobacillus spp*., such as *L. rhamnosus, L. plantarum*, and *L. acidophilus*, which are also referred to as lactic bacteria and play a role in the health of women microbiota by preventing pathogen growth.^57^ Among the vaginal fungal communities, *Candida spp*. is the most represented and is generally considered commensal in homeostatic conditions.^58^ However, in dysbiotic conditions, when the vaginal microbiota is imbalanced and a significant bacterial population is decreased, bacterial-fungal homeostatic interactions can be disorganized, resulting in yeast expansion and infection. Antibiotics, poor hygiene or contraceptive use are typical factors responsible for vaginal dysbiosis and are commonly related to vulvovaginal yeast infection onset, such as candidiasis, which is mostly caused by *C. albicans*, but other *Candida* species can also be responsible, such as *Candida glabrata* or *Candida tropicalis*.^59^ It is assumed that lactic bacteria are involved in yeast overgrowth control; therefore, their disappearance in the vaginal microbiota is considered a triggering event leading to yeast pathogen infections.^60^ Then, lactic bacteria appeared to be key actors in pathogen prevention in the vaginal microbiota, and various preclinical studies have evaluated the effect of lactic bacteria delivery to restore the vaginal microbiota and to manage yeast infections.^61–63^

Lactic bacteria are known to secrete diverse metabolites, such as hydrogen peroxide, antimicrobial compounds, and high concentration of weak organic acids, such as lactic and acetic acids. In the vaginal environment, organic acid secretion plays an important role in the maintenance of homeostasis.^64^ *In vitro* and *in vivo* studies have suggested that lactic bacteria secretion, and especially organic acids, are key compounds involved in the fight against fungal pathogen infections by inducing an acidic environment.^65^ In studies, the best inhibitory effect of lactobacilli strains on *Candida* growth is associated with the highest organic acid production.^62^ By acidifying the environment, lactic and acetic acids seem to be able to significantly prevent *Candida spp*. growth and therefore pathology onset. Findings have demonstrated that the inhibitory effects of organic acids are pH-dependent, as neutral medium cancels the effects of lactic and acetic acids.^66,67^ Jorgensen *et al*. suggested that a low pH prevents *C. albicans* from undergoing the yeast-hyphal transition, leading to energy consumption, growth inhibition and cell death.^62^ When chronically exposed to acidic stress, *C. albicans* exhibits a transcriptional shift, notably in iron homeostasis-involved genes. Interestingly, Cottier *et al*. reported, among various gene expression changes, an increase in genes involved in iron homeostasis *in vitro*, placing *C. albicans* in a metabolic state similar to starvation, with low transcription, translation and growth.^68^ However, the effects of these organic acids on fungi are controversial since several studies have shown opposite results. Indeed, inhibition of *Candida spp*. in coculture with vaginal lactic bacteria is not observed in some studies.^69^ *Candida spp*. are able to develop an adaptive response, inducing a tolerance to organic acids and low pH.^70^ Several mechanisms explain this tolerance occurrence, for example, ammonia production that neutralizes the environment and then decreases the effects of organic acid, the organic acid consumption ability, and a less permeable membrane to lactic acid.^71^ Consequently, there are still questions on the actual mechanisms involved in the lactic bacteria effect on *Candida* vaginal pathogenesis.

Indeed, studies from Ene *et al*. demonstrated that lactic acid could aggravate vaginal candidiasis by modifying the cell wall architecture of *C. albicans*.^72^ The carbon source in the environment strongly influences the yeast genome, inducing important alterations in the thickness and composition of the cell wall. When grown on lactate rather than glucose, such alterations appeared in *C. albicans*. The vagina is a glucose-limited environment (approximately 0.5%^73^), and lactobacilli supply a significant amount of lactic acid, which thus represents the main carbon source available for yeast. Ene *et al*. discovered a modulation of cell recognition by immune cells and cytokine production in response to lactate-grown *C. albicans* compared to glucose-grown *C. albicans*.^74^ By modifying its cell wall composition, lactate-grown *C. albicans* alters its interaction with innate immune cells, such as macrophages. These *Candida* cells are taken up less efficiently and escape more easily from macrophages. Moreover, IL-10 production was stimulated, and IL-17 production decreased. Overall, these results support the idea that a single metabolite, as is the case here with lactic acid, is capable of inducing strongly opposing effects, depending on the level of metabolites produced and probably other as yet unidentified parameters,^75^ such as physical and chemical parameters of the environment.

## Gut short chain fatty acids involvement in the bacterial-fungal relationship

Bacterial fermentation of indigestible fibers in the gastrointestinal tract (GIT) induces the production of short-chain fatty acids (SCFAs), commonly represented by their three more abundant constituents: acetate, butyrate and propionate.^76^ SCFAs are major metabolites in the gut, with the highest abundance found in the colon. Their concentration in feces is a prevalent marker used to evaluate the GIT health status and provides information about the gut microbiota equilibrium.^77,78^ These metabolites play a notably significant role in interactions between bacteria and fungi in the gut.

Gut SCFAs are not produced by all bacteria but by specific populations; consequently, a decrease in these bacterial populations, by antibiotics, for example, would be associated with a decrease in the SCFA abundance, influencing fungal growth. An *in vivo* experiment showed that cefoperazone treatment in mice leads to a loss of SCFA-producing bacteria, causing a reduced SCFA cecal concentration that facilitates *C. albicans* growth and colonization in the gut.^79^ Following these results, *in vitro* studies have shown that environmental SCFAs modulate *C. albicans* development. When cultured with physiological concentrations of acetate, butyrate and propionate, *C. albicans* shows defects in growth, filamentation and metabolic activity.^79^ *C. albicans* cultured with an antibiotic-associated SCFA concentration induces a minimal inhibitory effect on its growth; thus, it appears that the SCFA effect is dose-dependent. Based on these results, studies have hypothesized about the underlying mechanisms that could explain this SCFA inhibitory effect on *C. albicans*. SCFAs are related to an acidic environment; thus, pH lowering has been proposed to explain their suppressive effect. However, experiments analyzing low pH cultures, which correspond to the pH found in medium containing SCFAs, did not demonstrate any inhibitory effect on *C. albicans* growth.^79^ Other experiments showed that only acidic forms of SCFAs are able to impair the *C. albicans* population. Hence, it seems that a low pH is required to observe the negative impact of SCFAs but is not directly involved in growth inhibition. SCFAs are able to suppress histone deacetylation activity, which alters the expression of many genes in a wide range of cells, including fungi.^80^ Of the three major SCFAs, butyrate is the most efficient inhibitor of histone deacetylation, exhibiting a higher inhibitory activity than acetate and propionate. Research on the *in vitro* butyrate effect on several gut pathogenic fungi, such as *C. albicans, Candida parapsilosis* and *C. neoformans*, supports the hypothesis of growth inhibition driven by interference with histone deacetylase (HDAC) enzymes.^81^ Butyrate has shown a strong negative and dose-dependent effect on the biofilm formation of these three fungi, caused by *Candida* filamentation inhibition and *C. neoformans* capsule and melanin formation impairment.^81^

*C. albicans* filamentation and *C. neoformans* capsule formation are vital morphogenic virulence features regulated by HDAC activity; consequently, HDAC inhibition can attenuate fungal virulence. Due to its various virulence factors, *C. neoformans* presents high phenotypic plasticity. Its ability to produce a capsule and synthesize melanin, for example, are mechanisms that help this fungus to escape from the host immune system and thus facilitate its growth and colonization.^82,83^ In the presence of sodium butyrate, *C. neoformans* loses these attributes and is weakened, compromising its settlement in the host. *In vitro* experiments show that the HDAC inhibitory effect of butyrate interferes with *C. neoformans* growth, with a more pronounced impact at 37°C compared to 30°C cultures.^84^ Hence, by inhibiting the growth ability of *C. neoformans* at 37°C, butyrate affects its ability to colonize mammalian hosts. Concerning *C. albicans*, butyrate has shown an *in vitro* negative effect on filamentation, an important virulence factor for the fungus.^85,86^ Moreover, *C. albicans* filamentation defects caused by HDAC inhibition reduce yeast adhesion to host epithelial cells, another key step for disease pathogenesis.^87^ Therefore, SCFA-producing, and more precisely butyrate-producing, bacteria seem to have the capacity to impair the growth and virulence of pathogenic fungi through an HDAC inhibition mechanism.

Recently, a study established a link between bacterial-fungal interactions in the neonatal gut involving SCFA production and asthma development.^88^ This research follows a previous article, which demonstrated that, in addition to bacterial dysbiosis, fungal dysbiosis in the gut of 3-month-old Ecuadorian infants increases the risk of developing asthma at 5 years of age.^89^ This fungal dysbiosis is characterized by a general increase in the fungal population in the gut and, more particularly, an increased abundance of *Pichia kudriavzevii* (also known as *Candida krusei*). Furthermore, asthma occurrence is associated with reduced levels of fecal SCFAs, assigned to bacterial dysbiosis and certainly related to the loss of SCFA-producing bacteria. In light of these observations, Boutin *et al*. developed a mouse fungal dysbiosis model characterized by *P. kudriavzevii* overgrowth to determine the influence of fungal dysbiosis in early life on asthma outcome later in life. This model demonstrated that fungal exposure of mouse pups during the 2 weeks following birth, followed by airway inflammation at 6 weeks of age using the house dust mite protocol, induced increased lung inflammation in mice exposed to *P. kudriavzevii* in the neonatal period. To investigate the protective role of SCFAs against *P. kudriavzevii*-induced asthma development, cultures of *P. kudriavzevii* in medium containing physiological concentrations of acetate, butyrate and propionate at a colonic pH of 6.5 were conducted. SCFAs inhibited *P. kudriavzevii* growth and pseudohyphae formation, which consequently impaired *P. kudriavzevii’s* ability to adhere to intestinal epithelial cells and hence its ability to colonize the gut niche. These findings were confirmed by *in vivo* experiments, where antibiotic-treated mice administered *P. kudriavzevii* exhibited reduced fungal colonization when supplemented with a SCFA cocktail in their drinking water. The underlying mechanism of the SCFA inhibitory effect on *P. kudriavzevii* has not been elucidated at this time. The neonatal period is a crucial period during which microbiota and immune system development occur. Therefore, any disturbance during this period in the gut niche, a significant area where the microbiota and immune system communicate and mutually shape themselves, can induce serious consequences in later life. This study highlights a critical role of SCFA-associated bacterial–fungal interactions in early life and illustrates how impairment in these interactions may have a strong impact on homeostasis maintenance beyond the local effect and lead to long-term consequences, such as immune-related disease occurrence afterward.

SCFA production in the gut microbiota is mostly associated with the bacterial population. If fungi are able to synthesize fatty acids,^90^ there is no evidence of natural fungal competence to secrete SCFAs. However, some studies have observed an increase in the butyrate concentration when *Saccharomyces boulardii* is administered in a preclinical model, in association with *Lactobacillus rhamnosus* GG,^91^ and in patients under enteral nutrition.^92^ However, this butyrate release enhancement seems to be more induced through bacterial stimulation by *S. boulardii* rather than through *S. boulardii* secretion itself. These studies nevertheless point out a potential therapeutic approach exploiting synergetic interactions between bacteria and fungi. Such a synergetic interaction was illustrated by Roussel *et al*. with their paper concluding the beneficial effect of *S. cerevisiae* administration in reducing the pathogenicity of enterotoxigenic *E. coli* (ETEC).^93^ Using an *in vitro* human gut model in the presence of complete microbiota (M-SHIME), researchers demonstrated that *S. cerevisiae* attenuated the virulence of ETEC. This effect was first explained by the possible cross-feeding between *S. cerevisiae* and beneficial bacteria of the gut microbiota. Indeed, this study showed an increase in the population of SCFA-producing bacteria (*Faecalibacterium, Roseburia*, and *Bifidobacterium*) and in the SCFA concentration (butyrate and acetate) after *S. cerevisiae* administration, potentially induced by the bacterial use of yeast α-mannans as a substrate. However, Roussel *et al*. also observed the disruption of membrane integrity of ETEC following *S. cerevisiae* supplementation. Several hypotheses have been proposed to explain the effect of *S. cerevisiae* on ETEC membrane depolarization: (i) the increase in ethanol production with *S. cerevisiae* treatment could induce *E. coli* membrane disruption, as previously shown;^94^ and (ii) the ability of *S. cerevisiae* to deconjugate bile acids, which are well-known metabolites involved in membrane solubilization.

This latter hypothesis highlights an interesting and poorly documented metabolic interaction centered on the bacterial and fungal competence to deconjugate bile acids and its consequences on their microbial neighbors. Roussel *et al*. speculated that fungi are capable of inducing negative effects on bacterial viability through the transformation of bile acids, and that bacteria, also able to form secondary bile acids,^95^ could therefore impact the fungal population. One study showed the changes induced by lithocholic acid and deoxycholic acid, two secondary bile acids derived from bacteria, on the growth and morphogenesis of *C. albicans*.^96^ Unfortunately, studies regarding the role of deconjugated bile acids in bacterial-fungal interactions are limited, despite the obvious relevance of this research topic for human health.

## Multiple effects of fungal ethanol on bacteria

Ethanol is a well-known alcohol produced by fungal or bacterial sugar fermentation.^97^ The food industry commonly uses yeast in alcoholic beverage production processes, highlighting fungal fermentation efficiency in comparison to bacteria. Through this high capacity to produce ethanol in their environment, various ethanol-driven effects of fungi on bacteria have been reported, depending on the ethanol concentration. An ethanol concentration of 5% or more usually inhibits growth or kills bacteria, while a physiological ethanol concentration ranging between 0.1 and 1.1%, similar to the concentration found in the microbial community, induces significant changes in bacterial biological activities.

In experiments conducted to observe *in vitro* yeast and bacteria interactions, Smith *et al*. noted a synergistic relationship between *S. cerevisiae* and *Acinetobacter* bacteria, two organisms frequently coexisting in various environments, including humans, where they can both become pathogens. With several tests, they elucidated that this beneficial interaction was mediated by a diffusible factor, a small organic molecule produced by *S. cerevisiae*, and identified this molecule as ethanol.^98^ This study showed a beneficial effect of *S. cerevisiae* ethanol on *Acinetobacter* bacterial growth, notably on *A. baumannii* and *A. haemolyticus*, two pathogenic bacterial species. Several strains of *S. cerevisiae* were studied. Laboratory strains are generally used for *in vitro* experiments, but wild isolates are also collected in diverse natural environments (soil, water, plants and animals). Among all tested strains, the strongest growth effect was induced by *S. cerevisiae* strains found on patient hospital pillows, which demonstrated the most efficient ethanol production, revealing a dose-dependent effect of ethanol. *Acinetobacter* bacteria are anaerobic and consume several carbon sources; however, few species use glucose.^99^ *Acinetobacter* are therefore dependent on yeast to convert sugar into ethanol, an easier carbon source for them to consume.^100^ Beyond its potential use as a carbon source, S. *cerevisiae* ethanol leads to other advantages for *Acinetobacter* strains. Indeed, the presence of ethanol in the culture medium of *Acinetobacter* stimulates their salt tolerance and increases their virulence against their natural predator, the *C. elegans* worm, in contrast with bacteria fed with other carbon sources. Hence, ethanol may operate by signaling pathways involved in specific stress tolerance and virulence. Further studies pointed out an ethanol influence on *A. baumannii* iron assimilation, the phosphate transport system and an enhancement of phospholipase C secretion, an enzyme related to virulence in *A. baumannii*,^101^ which could partly explain how fungal ethanol impacts *A. baumannii*.

Other studies have focused on ethanol-driven interactions between fungi and bacteria and have investigated alternative mechanisms involved in these interactions. A study from Chen *et al*. reported a stimulatory effect of ethanol produced by *C. albicans* on *P. aeruginosa* biofilm formation.^102^ These observations have been associated with the signaling pathway c-di-GMP (cyclic-di-guanosine monophosphate) in *P. aeruginosa*, which is related to biofilm formation.^103^ High levels of c-di-GMP lead to increased matrix production and decreased flagellar motility, causing biofilm development in *P. aeruginosa*.^104,105^ This ethanol effect seems to be linked to WspR, a diguanylate cyclase enzyme involved in c-di-GMP formation and activated through a specific membrane sensor, WspA.^106^ To confirm the role of the WspR enzyme in the ethanol effect, *in vitro* experiments demonstrated the absence of a reaction to ethanol in the WspA mutant *P. aeruginosa*. The authors hypothesized that there was ethanol-induced WspR system alteration, suggesting that ethanol could increase membrane rigidity, causing a modification in the fatty acid composition of the membrane. The WspA sensor could therefore be activated by this modification in lipid composition or in membrane physical properties. Interestingly, cystic fibrosis patients differ from healthy patients by higher breath exhaled ethanol levels, confirming the relationship among *C. albicans* ethanol production, *P. aeruginosa* virulence and cystic fibrosis occurrence.^107^ Another mechanism of ethanol-induced stress tolerance is the stimulation of trehalose production in *P. aeruginosa*.^108^ Trehalose is a disaccharide involved in diverse biological activities, such as protection against environmental stresses (osmotic, oxidative, heat and cold) through protein stabilization and reduction of denatured protein aggregate formation, and can be used as a carbon source.^109^ Additionally, these two mechanisms explain the potential role of ethanol in exopolysaccharide overproduction by *P. aeruginosa* and its involvement in biofilm formation and mucoidy. The exopolysaccharide alginate (linked to trehalose production^108^) and the Pel operon (linked to WspR activation^102^) are both correlated with mucoid state conversion of *P. aeruginosa*, a pathogenic characteristic generally observed in cystic fibrosis isolates and associated with a decline in lung function.^110^ Moreover, it is relevant to note that ethanol seems to interact with the QS system in *P. aeruginosa*. Ethanol stimulation of trehalose involves acylhomoserine-lactone (AHL), a well-known *P. aeruginosa* QS molecule.^108^ Phenazines, i.e., pyocyanin, are *P. aeruginosa* secreted factors necessary for pathogenicity and are involved in bacterial QS.^111^ When exposed to low levels of phenazines, *C. albicans* increases its ethanol production;^112^ furthermore, it has been observed that phenazine production is altered in the presence of ethanol.^102^ Additionally, nutrient availability in the environment influences the interaction between *C. albicans* ethanol and *P. aeruginosa* phenazines production. Doing *et al*. demonstrated a conditional stimulation of *P. aeruginosa* phenazines production in response to *C. albicans* ethanol, depending on phosphate availability.^113^ Under phosphate-limited conditions, ethanol activates PhoB, a *P. aeruginosa* response regulator involved in phosphate deficiency conditions, which enhances phenazine production and motility.^114^ These observations once again demonstrate that bacterial-fungal metabolic processes cross-interact in multiple ways, resulting in a complex network involving diverse actors or parameters and inducing various consequences on the host that are not yet fully understood.

Recently, an intriguing response from bacteria to yeast ethanol production has been discovered. To reduce yeast glucose consumption and ethanol production, bacteria secrete a prion named [*GAR^+^*], which inhibits glucose intake in *S. cerevisiae* through hereditary genetic manipulation of yeast metabolism.^115^ In natural environments, in the presence of glucose, yeast switch off metabolic pathways involved in the use of other carbon sources and prioritize glucose consumption.^116^ This repression system occurs in several organisms but is extremely rigorous in *S. cerevisiae*. [*GAR^+^*] prion, designated as such because it overcomes the “glucose-associated repression”, is a proteic element found in wild strains of yeast, allowing them to diversify their phenotypes.^117^ Prions are also molecules involved in social interactions between organisms that shape the communal dynamic in accordance with the environment. This [*GAR^+^*] interaction described between bacteria and *S. cerevisiae* provides strong adaptive benefits for both organisms. Natural environments are rarely composed of a single carbon source but rather are composed of mixed glucose and other carbon sources. Therefore, by multiplying carbon source options, yeast can increase their fitness and lifespan in such environments. By reducing glucose consumption in yeast, bacteria also take advantage of this interaction by minimizing fermentation and thus ethanol production, which provides them with a more suitable environment. Among the wide range of tested bacteria, 30% have demonstrated an ability to induce [*GAR^+^*] in yeast. These bacteria can be either gram-positive or gram-negative bacteria, with no genus or species clustering, indicating that this ability to produce [*GAR^+^*] prion is widely disseminated in the bacterial kingdom.

## Amino acid and vitamin exchange among bacterial-fungal communities

A characteristic of microbial communities is nutrient sharing, as amino acids and vitamins are essential for various biological processes.^118,119^ Both bacterial and fungal kingdoms are able to synthesize these metabolites and diffuse them in their niche, providing counterparts to absorb them. However, a naturally or genetically induced lack of one or more biosynthetic pathways is frequently observed in these microorganisms, making them “auxotrophic”. Thus, they depend on an exogenous supply of amino acids and vitamins from their environment or nearby producing cells. The ecosystem strongly influences biosynthetic pathway mutations.^120,121^ Metabolite richness in the environment is associated with the loss of biosynthetic pathway genes, and organisms favor metabolite uptake rather than the energy-intensive production of complex molecules, such as amino acids or vitamins.^120^ This metabolic adaptation acts like a natural selection, which supports mutations inducing the loss of no longer required functions, often energy-consuming, since metabolites are available in the environment. In the microbial community, i.e., biofilm, metabolite exchange between auxotrophic and prototrophic cells commonly occurs in a process called cooperative cross-feeding, where co-living cells find benefits in sharing their metabolites with each other.^122,123^ This metabolic cooperation allows for survival in environments where metabolite availability fluctuates over time. In *S. cerevisiae* colonies, amino acid prototrophic yeast show a preference for using environmental amino acids rather than synthesizing them themselves.^124^ This phenomenon occurs in cohabiting yeast, which illustrates the evolution toward a behavior characterized by an energy-saving mechanism, conferring to the yeast a high metabolic flexibility and adaptability. Among studies on amino acid and vitamin cross-feeding interactions, most are based on bacteria, and a few explore yeast interactions, whereas bacterial-fungal amino acid and vitamin exchange studies are almost nonexistent. Currently, significant attention is given to interspecies (bacterial or yeast) cross-feeding interactions; however, research on amino acid and vitamin exchange between bacteria and fungi is limited and deserves more consideration. Although limited, there are few data on these fungal-bacterial interactions in the literature. A study reported a symbiotic interaction based on amino acids released by *S. cerevisiae* that benefit lactic acid bacteria.^125^ Under specific conditions, in this case, nitrogen overflow, *S. cerevisiae* secretes high amounts of amino acids to its environment. This mechanism helps the yeast discharge excess intracellular nitrogen, notably amino acids, since high levels of nitrogen are harmful to the yeast. Thus, waste products from *S. cerevisiae* are assimilated by lactic acid bacteria and provide them with a significant supply of essential nutrients, reflecting a beneficial interaction. Interestingly, a study demonstrated an increased amino acid concentration and high rates of *P. aeruginosa* isolates that are auxotrophic for amino acids in cystic fibrosis patients’ lungs.^126^ We have seen in this review numerous examples of *P. aeruginosa* and *C. albicans* metabolic interactions related to the occurrence and severity of this disease. Moreover, nitrogen seems to play a role in cystic fibrosis severity.^127^ Hence, it would be interesting to investigate whether *C. albicans* is involved in amino acid enhancement associated with cystic fibrosis, potentially through a nitrogen mechanism, similar to the one observed in *S. cerevisiae*.

Vitamins, and notably B vitamins, are necessary for several biological processes in prokaryotic and eukaryotic cells. As expected, most of the articles analyzing vitamin sharing in microbial communities focus on bacteria, and fungi are understudied. However, studies of bacterial vitamin exchange demonstrate the high ability of microbes to interact and cooperate dynamically,^128,129^ which can then be extended to mixed and more complex bacterial-fungal communities, where such interactions undoubtedly occur. One of the rare studies reporting a bacterial-fungal interaction involving vitamins demonstrated a biotin-centered (vitamin B7) interaction between *Penicillium* spp. and *E. coli*.^15^ The results indicated that *E. coli*, a biotin prototroph bacterium, upregulates biotin biosynthesis genes in the presence of *Penicillium*, which suggests a bacterial increased requirement for biotin when cohabiting with this fungus. The authors interpreted this observation as an increased need for biotin synthesis for the bacterium, induced by competition between bacteria and fungi for available biotin in the medium. Consequently, it seems that this work demonstrated a potential rivalry between the two organisms for environmental biotin. However, it would be interesting to examine the evolution of cogrowing *Penicillium* and *E. coli* and to follow how both organisms adapt to low biotin availability, which could potentially develop metabolic cooperation around biotin. *C. albicans* is a biotin auxotroph and thus requires exogenous biotin in its environment to grow and become virulent. Indeed, germ tube formation in *C. albicans* correlates with the biotin concentration in the medium,^130^ and the biotin uptake system in yeast is critical for the survival of macrophages and the induction of systemic infection.^131^ Another vitamin that is crucial for *Candida* spp. survival in macrophages is thiamine, or vitamin B1, used by prokaryotic and eukaryotic cells for its antioxidative properties. *C. albicans* exposed to reactive oxygen species exhibited a higher exogenous thiamine use.^132^ However, in a thiamine-free medium, an impairment of *C. albicans* growth is observed when faced with oxidative stress. If *C. albicans* is able to synthesize thiamine, it appears that secreted levels are too low to induce a protective effect against oxidative stress and that the yeast might need an exogenous thiamine supply for defense, presumably from coliving organisms.

## Conclusion

**Figure 1.**
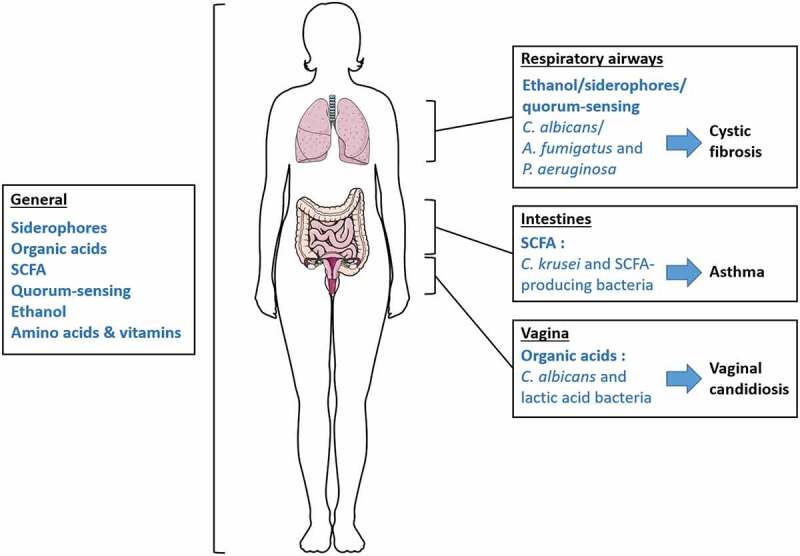
Summary of relevant bacterial-fungal metabolic interactions discussed in this review. Bacteria and fungi generally interact through different forms of metabolic crosstalk in the human body. However, some metabolic interactions occur in specific organs and are associated with human health and disease onset. Studies on the bacterial-fungal metabolic interactions in respiratory airways mainly focus on the fungi A. fumigatus and C. albicans, and their distinct relationships with the bacterium P. aeruginosa, although these relationships involve several metabolic pathways.

**Figure 2.**
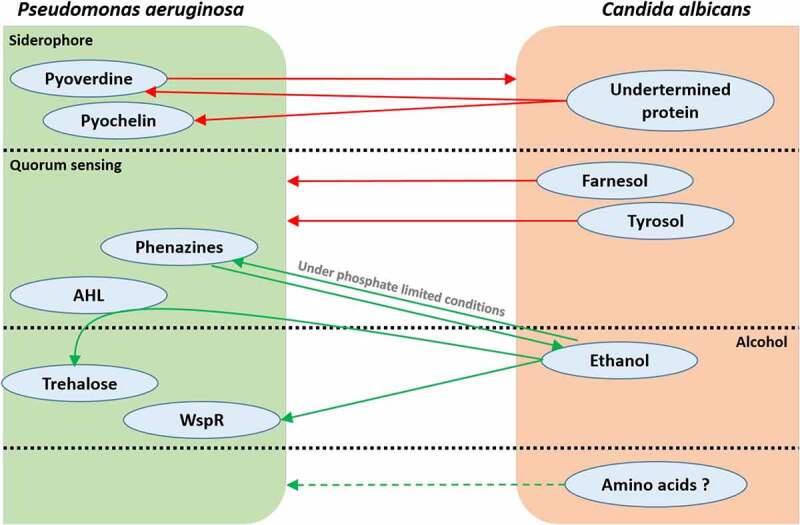
The multiplicity of metabolic interactions between P. aeruginosa and C. albicans. Green arrows represent a metabolite-driven enhancement of growth and/or metabolite secretion. Red arrows represent a metabolitedriven inhibition of growth and/or metabolite secretion. Quorum-sensing and alcohol communication pathways cross-interact with each other, as seen with the role played by P. aeruginosa HSL in fungal ethanol-driven stimulation of bacterial trehalose production. Amino acids are hypothetically involved in P. aeruginosa/C. albicans communication; however, this hypothesis has not been studied yet.

The term ecosystem designates coliving organisms in a defined environment that interact with each other, a definition completely representing the relationship found in human microbiota. These interactions shape microorganism communities through symbiotic and antagonistic behaviors, notably using metabolites as elements for communication. Two studies on colorectal cancer have demonstrated modifications in the fungal microbiota community and in the networks of inter-kingdom interactions suggesting a possible link between disease and the equilibrium of these two microbial communities.^133,134^ However, the mechanisms behind these interactions are not yet fully understood, but can be the results of a complex exchange of metabolites. Although, specific bacterial-fungal metabolic interactions have been related to the occurrence of pathologies in several studies. Cystic fibrosis, infant asthma and candidiasis correlate with particular bacterial-fungal communities, supporting a significant role of these interkingdom interactions in human disease onset (Figure 1). Other documented metabolic interactions have an impact on specific strain virulence, potentially related to pathology development, but also on beneficial strains, which could enhance health. Most publications referring to bacterial-fungal interactions focus on their influence on pathogenic organisms and on the development of pathologies and rarely on their role in maintaining health. More research on how bacterial-fungal crosstalk influences and maintains homeostasis is needed to demonstrate the significant role of these interactions in health. However, knowledge about pathogens can be used to extrapolate to nonpathogenic microorganisms and can serve as an initial research direction focused on ecosystem balance. Bacteria and fungi are able to metabolically interact by several molecules or metabolic pathways, inducing various effects on the growth of their counterparts. The example of the bacterium *P. aeruginosa* and the fungus *C. albicans* interactions described throughout this review denotes that these two organisms communicate through multiple metabolite activities, resulting in various, beneficial or deleterious, responses for both protagonists (Figure 2). Moreover, some pathways influence others, as we have seen with the *P. aeruginosa* QS role in the trehalose pathway in response to *C. albicans* ethanol production, which demonstrates intricate connections and further complexifies the observed interactions.

It is important to note the need to improve laboratory techniques with the aim of enhancing bacterial-fungal metabolic interaction studies. Current methods to analyze such interactions between microorganisms commonly use synthetic media, where nutrients are highly available and culture time is generally limited to a few hours or days. Such protocols are far from the reality of the natural environments that organisms inhabit. Organisms need time and a specific environment to develop and evolve together to an “open to dialog” community. Natural environments usually undergo fluctuating nutrient availability, which forces organisms to adapt, sometimes in a competitor or mutualistic relationship. Studies on *in vitro* system amelioration have moved forward; however, if they are getting closer to the actual natural conditions,^135^ further work is still necessary to reproduce an identical environment. The development of new *in vitro* approaches opens the way to alternatives closer to reality for microorganisms’ interactions studies. The “organ-on-chip” technology provides opportunities to reach more realistic and natural co-culture environments. However, this technique is still in development and is currently used to identify host-microbiota interactions, and underutilized for microorganisms’ interactions in human. One study has applied the organ-on-chip technology to define bacteria and fungi interactions in the soil microbiota.^136^ Nevertheless, these researches demonstrate the potential of this process to determine how bacteria and fungi communicate and lay the foundation for further bacteria/fungi interactions researches in human. In the recent years, major advances have been provided to improve this new microfluidic method, on the means to modulate oxygen in the environment for example,^137,138^ an necessary development to investigate anaerobic microorganisms interactions in human. Gnotobiotic *in vivo* models also offer an interesting alternative to *in vitro* studies, in the sense that the environment where inoculated organisms evolve is more similar to their natural environment in terms of nutrients and physiological properties, thus representing interactions that are more realistic.^139,140^

Currently, only a small fraction of human inhabiting organisms is cultivable in the laboratory,^141,142^ which means that a wide number of bacteria and fungi are still unstudied. The reasons for the difficulties in their culture are highly related to the challenge of reproducing an identical environment to that in which they usually live. We can also hypothesize that some microorganisms might require, for their growth and survival, the presence of others through some of the metabolic exchanges we described in this review or other yet undescribed ones. Thus, synthetic media and monocultures are presumably inadequate for cultivating such demanding strains. Hence, the gnotobiotic model and coculture with several naturally found coliving strains, potentially from different kingdoms, could facilitate the discovery of original and relevant strains, as well as specific growth conditions and/or multicrossing interactions involving a variety of species that intensify potential exchanges between organisms, which could ultimately be related to human health. Presently, studies only scratch the surface of the bacterial-fungal interactions. We barely appreciate the importance of the impact they represent to our health. Therefore, microbiota-focused studies on health enhancement and on new therapeutic strategies should give more consideration to the significance of bacterial-fungal interactions in human health.

## Notes

Data sharing is not applicable to this article as no new data were created or analyzed in this study.
